# A LiDAR-Camera-Inertial-GNSS Apparatus for 3D Multimodal Dataset Collection in Woodland Scenarios

**DOI:** 10.3390/s23156676

**Published:** 2023-07-26

**Authors:** Mário P. Cristóvão, David Portugal, Afonso E. Carvalho, João Filipe Ferreira

**Affiliations:** 1 Institute of Systems and Robotics, Department of Electrical Engineering and Computers, University of Coimbra, 3030-290 Coimbra, Portugal; 2 Department of Electrical Engineering and Computers, University of Coimbra, 3030-290 Coimbra, Portugal; 3 Computational Intelligence and Applications Research Group, Department of Computer Science, School of Science and Technology, Nottingham Trent University, Nottingham NG11 8NS, UK

**Keywords:** multi-sensor apparatus, multimodal dataset collection, forestry robotics, LiDAR, inertial measurement unit, depth cameras, GNSS

## Abstract

Forestry operations have become of great importance for a sustainable environment in the past few decades due to the increasing toll induced by rural abandonment and climate change. Robotics presents a promising solution to this problem; however, gathering the necessary data for developing and testing algorithms can be challenging. This work proposes a portable multi-sensor apparatus to collect relevant data generated by several onboard sensors. The system incorporates Laser Imaging, Detection and Ranging (LiDAR), two stereo depth cameras and a dedicated inertial measurement unit (IMU) to obtain environmental data, which are coupled with an Android app that extracts Global Navigation Satellite System (GNSS) information from a cell phone. Acquired data can then be used for a myriad of perception-based applications, such as localization and mapping, flammable material identification, traversability analysis, path planning and/or semantic segmentation toward (semi-)automated forestry actuation. The modular architecture proposed is built on Robot Operating System (ROS) and Docker to facilitate data collection and the upgradability of the system. We validate the apparatus’ effectiveness in collecting datasets and its flexibility by carrying out a case study for Simultaneous Localization and Mapping (SLAM) in a challenging woodland environment, thus allowing us to compare fundamentally different methods with the multimodal system proposed.

## 1. Introduction

Forest and woodland maintenance are crucial and challenging tasks that require monitoring forests, planting trees, and removing invasive species. These tasks can be physically demanding and time-consuming for human workers, posing significant safety risks. While autonomous robots have the potential to revolutionize forestry maintenance, the existing technology has limitations that prevent widespread adoption. One of the most important of these tasks, landscape maintenance, has become particularly relevant as forest fires have become increasingly prevalent in recent decades.

Current forestry robots [1,2] often lack the flexibility needed to easily navigate through complex and dynamic forest environments, making data acquisition slow and cumbersome. Forest areas are characterized by various obstacles, such as dense vegetation, uneven terrains, and dynamic changes due to growth and decay. Conventional forestry robots may struggle to maneuver through these challenging conditions, leading to limited coverage and incomplete data acquisition [3]. The lack of flexibility in conventional forestry robots also hampers their ability to access hard-to-reach areas.

**Figure 1:**
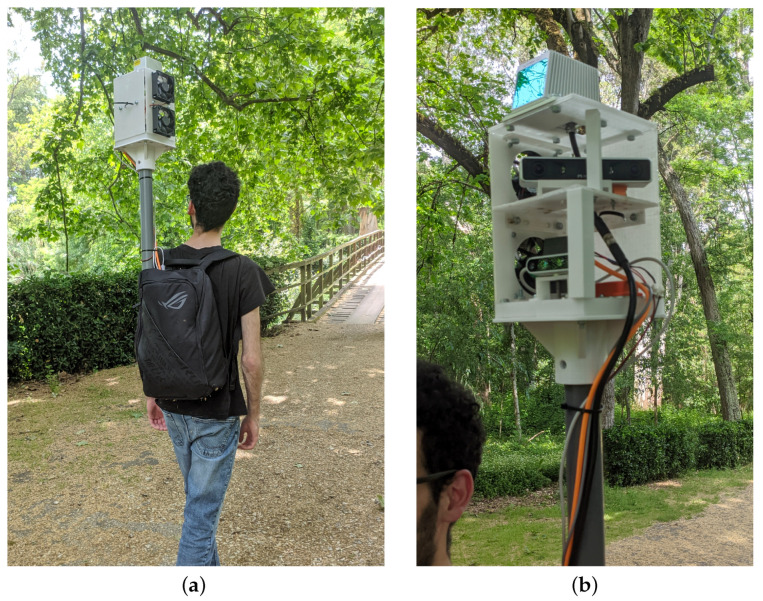
Illustration of the LiDAR-Camera-Inertial-GNSS apparatus proposed for dataset collection. (**a**) Operator carrying the apparatus backpack. (**b**) Close-up view of the sensors.

To overcome existing limitations, this paper proposes the development of a lightweight and portable LiDAR-Camera-Inertial-GNSS apparatus with an onboard computer for acquiring datasets in forests and woodlands. The apparatus, illustrated in Figure 1, collects multiple sensor modalities such as accelerometer, gyroscope and magnetometer data from an Inertial Measurement Unit (IMU), RGB and 3D Depth information from two cameras, 3D Light Detection and Ranging (LiDAR) scans, and Global Navigation Satellite System (GNSS) information to create detailed and accurate datasets of forest environments.

The apparatus aims to solve the critical challenge of data acquisition, facilitating the planning, testing and deployment of autonomous robots for forestry maintenance, and it has key potential benefits, including improving the safety and efficiency of acquiring datasets and reducing costs associated with deploying an automated vehicle in the field. This paper also presents an experimental evaluation of the system in a real forest environment, where Simultaneous Localization and Mapping (SLAM) implementations have been tested as a case study, demonstrating the feasibility, flexibility and potential of the system proposed.

By proposing a lightweight and portable multi-sensor apparatus, this study provides significant contributions to the field of forestry robotics, such as a publicly available ready-to-use dataset [4] that enables researchers to analyze forest environments in depth, and developing and testing perception-based methods with real-world data. In addition to the apparatus design description and important lessons learned, the architecture developed to record and store datasets also represents an important contribution, bringing a novel, modular, and user-friendly architecture for acquiring multisensory outdoor datasets, allowing different sensor configurations to be used with minor adjustments. Moreover, we also contribute with an in-house developed Android App for easily exposing smartphone GNSS data with ROS for use in Robotics [5]. As such, this work has the potential to pave the way for a more widespread use of autonomous robots in forests and woodland scenarios.

## 2. Use Case Scenario

Simultaneous Localization And Mapping, commonly referred to as SLAM, is the action of progressively building a map of an environment perceived by a moving entity (e.g., a robot) while persistently localizing in that map as the entity moves through space [6,7]. SLAM algorithms play a crucial role in enabling autonomous robots to navigate and perceive unknown environments in real time. However, the complex and dynamic nature of forest environments presents particularly significant challenges, making it essential to evaluate and refine SLAM methods offline using realistic datasets.

Below, we formulate a potential use case scenario to clarify our motivations and demonstrate how the collection of datasets can be used to test methods for artificial perception in Forestry Robotics as well as for localization and mapping in particular:

*In this use case, a research engineer undertakes a data collection mission in a remote forest area using an in-house developed multi-sensor apparatus. The mission aims to gather a diverse range of multimodal data, such as LiDAR, IMU, Depth and RGB cameras, and GNSS, to support research efforts.*

*He follows a systematic circular route through the dense vegetation of the forest while equipped with the multi-sensor apparatus worn as a backpack. The LiDAR sensor accurately measures the three-dimensional structure of trees and vegetation, while the IMU tracks the backpack’s orientation, including magnetic heading. Depth cameras and RGB images provide visual details of the forest, and GPS records positioning data throughout the entire journey.*

*Upon completing the route and returning to the starting point, he concludes the data collection process and disconnects the apparatus. Before sharing the data, he transfers the acquired dataset to his laptop as a ROS bag file and performs initial assessments as well as functional checks to ensure the quality of the dataset.*

*The shared dataset becomes a valuable resource for the Forestry Robotics community. For instance, a PhD student utilizes the collected data to test and refine SLAM algorithms, aiming to improve the precision and efficiency of robots in mapping and navigating forest environments, and contributing to the progress of forestry management and conservation practices by harnessing technological advancements.*

## 3. Background and Related Work

In this section, we start with an analysis of previous multi-sensor apparatuses designed for dataset collection, including those specifically tailored for SLAM and forestry applications, followed by a review of seminal work on 3D SLAM for a better understanding of existing state-of-the-art methods.

In the past, a few portable and light sensing apparatuses designed to collect data from the environment around us have already been proposed. To the best of our knowledge, Oveland et al. [8] was the first to develop a portable apparatus to be used in a forest environment. They compared different methodologies to study the Diameter at Breast Height (DBH), an important feature in forestry inventory, reaching the conclusion that a portable apparatus with multiple sensors, such as LiDAR and an IMU, is a viable alternative to perform forest inventory. In Proudman et al.’s work  [9], a portable system was designed for estimating the DBH of trees in forestry applications. However, their choice of using a metal stick instead of a backpack introduces the issue of user fatigue. This design may lead to excessive variations in stick position, resulting in unintelligible and uncontrolled movements, which can negatively impact data collection. While their system had the benefit of a built-in display for real-time data visualization, the design limitation raises concerns about the accuracy and consistency of the measurements. On the other hand, Su et al. [10] and Xiao et al. [10] developed an accurate backpack system with two orthogonally positioned LiDARs. Their backpack design overcomes the user fatigue issue associated with the metal stick approach, allowing for more stable movements during data acquisition. Since Su et al. aimed to measure DBH, their paper lacks effective metrics to assess the precision of localization. In contrast, Xiao et al. present the Relative Translation Error metric, introduced in [11], but they lack RGB-D and stereo information. Jelavic et al. developed a system for forestry-harvesting missions [2]. Their system aims to acquire a dataset to generate an *a priori* map of the deployment location for an autonomous harvesting excavator. While their implementation shares similarities with the present study by providing metrics to evaluate the precision of the SLAM algorithm used, it falls short in incorporating RGB-D and GNSS information. Sier et. al [12] designed a very complete apparatus on top of a cart wheel with the objective of comparing the performance of six different LiDARs in GNSS denied environments. LiDARs with both spinning and solid-state technologies were considered as well as a stereo fish-eye camera. The authors compare different state-of-the-art SLAM methods with different LiDAR configurations to assess the most appropriate combination. For forest environments, the authors concluded that the most robust combinations are the FAST-LIO [13] implementation using the more precise Ouster spinning LiDARs and the Livox Horizon using a LIO-based SLAM design for the Livox Horizon. In a recent study conducted by Faitli et al. [14], a new measurement setup was developed to collect LiDAR and IMU data for localization and mapping using a LIO-SAM-based method. While the system design shares similarities with the previous work of Proudman et al. [9], Faitli et al. focused more on evaluating the performance of their SLAM algorithm specifically designed for forest environments. However, it is important to note that their dataset did not include RGB information, which restricts the potential applications of their dataset. Another recent study by Li et al. [15] presented a new sensing kit that collected LiDAR-IMU datasets in multiple GNSS-denied scenarios, including a forest environment. Instead of using a backpack or a handheld design, the authors chose to develop a helmet that integrated the sensors, such as LiDAR, IMU, and GNSS, while storing the rest of the hardware in a backpack. According to the authors, the motion characteristics of the helmet approach were similar to those found in the handheld counterpart, involving quick shifting and shaking, whereas the backpack design only accounted for quick shifting. However, the level of fatigue that would result from supporting a 1.5 Kg load on top of the operator’s head remains uncertain. Once again, the datasets produced in this study lacked RGB-D information, which hinders the potential use cases of the collected datasets.

One of the most popular implementations of SLAM is Real-Time Appearance-Based Mapping (RTAB-Map) [16]. RTAB-Map is a graph-based SLAM system that relies on an image loop closure detector, offering several options for the back-end, namely GTSAM (default) [17], g2o [18] and TORO [19]. The loop closure detector uses a bag-of-words approach to determine the likelihood that a new image was taken from a previous or a new location. It can estimate odometry from IMU and wheel encoders, but it also supports Visual and LiDAR odometry as optional odometry sources. When executing loop closure, RTAB-Map reuses the features that were previously matched in Visual or LiDAR Odometry, which improves the overall performance. RTAB-Map can generate both 2D and 3D Occupancy grids.

Several LiDAR-based methods derive from LiDAR Odometry And Mapping, which is commonly known as LOAM. Although LOAM can create highly accurate maps, it usually performs poorly in places with few landmarks, such as long corridors. LeGO-LOAM [20] adds two additional modules to the LOAM technique: point cloud segmentation and loop closure. These extra components allow an improvement in computing performance and drift reduction over long distances but does not improve the results when used in a featureless environment. LeGO-LOAM uses the naive ICP algorithm to perform loop closure, but a more robust approach based on a point cloud descriptor is implemented in SC-LeGO-LOAM [20,21]. To help improve the performance in a low features environment, researchers have been recently adding an IMU to similar systems in a tightly coupled approach (see [13,22,23]), giving rise to the term LiDAR Inertial Odometry (LIO). For instance, the LIO-SAM [24] approach proposes a tightly coupled LiDAR framework atop of a factor graph. The implementation considers four different factors, namely IMU preintegration, LiDAR odometry, GPS and a loop closure factor, making it ideal for multi-sensor fusion and global optimization. Lately, several methods based on similar principles have been proposed [25,26,27,28,29,30,31,32].

Cartographer is Google’s implementation to solve the SLAM problem [33]. It is also a LiDAR-based graph SLAM divided into two main components: local SLAM (the front end) and global SLAM (the back-end). This approach takes input of a range-finding sensor, e.g., a LiDAR, and applies a band-pass filter to the input data. IMU can also be used to help figure out the robot rotation and to provide information on gravity direction, which is used in the 3D variant.

In order to evaluate SLAM systems, vital metrics such as Relative Translation Error (RTE) and Absolute Position Error (APE) were introduced in [11]. RTE measures the accuracy of estimating the relative translation between two positions. If the two positions are taken from the same location, a lower RTE implies a more precise localization estimate. On the other hand, APE quantifies the accuracy of absolute position estimation by comparing the estimated positions with ground truth values. A smaller APE indicates better localization accuracy.

The systems reviewed in this section provide valuable insights into the challenges and opportunities in the development of a portable apparatus for forestry applications. Table 1 presents a comparison between the reviewed systems and the SLAM algorithms tested using the data collected with each of these frameworks. Some systems focus on the forest application inventory, and other applications are particularly focused on determining the DBH of trees. Key metrics are missing in some works, making it difficult to effectively evaluate their performance for SLAM. The majority of the apparatuses reviewed in this study lack RGB-colored images of the environment. RGB information plays a crucial role in various artificial perception algorithms and methods, and its absence limits the potential use cases for both the apparatus and the dataset it generates. It is imperative to include RGB information in recorded datasets to enable a wider range of use cases. Furthermore, these systems are typically expensive, which is primarily due to the high costs associated with the prevalent LiDAR technology incorporated in them.

An estimate of the overall value of the above-mentioned works has been included in Table 1. While some costs are provided by the authors, most are estimated based on the current unit price of the sensors that make up the various apparatus systems.

By building upon the lessons learned from previous works, we have built an apparatus that combines multiple sensor modalities into a single lightweight, inexpensive and portable backpack system. The integration of multiple sensors allows for comprehensive data acquisition, which in turn enables a detailed and accurate perception of the forest environments. The apparatus presented in this study overcomes the limitations of prior systems by providing a wider range of sensory information. Additionally, the collected datasets are made publicly available, including high accuracy pose estimation off the shelf, with applicability potential beyond localization and mapping algorithms, as we demonstrate later on.

## 4. System Description

Among the reviewed approaches, the use of a wheel cart system, as demonstrated in [12], offers the most ergonomic solution. However, this approach significantly hampers the maneuverability of the apparatus. Forest environments present several challenges and obstacles such as uneven terrain, rocky surfaces, and dense vegetation, making wheeled vehicles generally impractical for navigation. On the other hand, mounting the entire system on a metal rod held firmly in the operator’s hands greatly enhances maneuverability, facilitating precise pointing and feature capture. Nonetheless, this approach comes with its limitations. The human operator may experience fatigue over time, resulting in reduced stability when holding the system and ultimately affecting the quality of the dataset.

The backpack method strikes a balance between ergonomics and maneuverability. It offers easier portability and improved stabilization during extended distances compared to the rod approach while remaining highly adaptable to uneven terrains. Taking into account the unique demands of forestry scenarios, the backpack method emerges as the most practical compromise, enabling operators to navigate through the challenging environments while maintaining stability and minimizing fatigue. Therefore, as seen in Figure 1, we decided on a backpack-based design for the apparatus proposed.

Our objective emphasizes the importance of collecting datasets that encompass a diverse range of sensory information from different sensor types. In mapping applications, accurate depth sensors play a crucial role, with LiDAR being widely recognized as the predominant sensor in this field. By incorporating an RGB-D camera, the datasets we generate become more versatile and can be utilized for various applications, such as segmentation and fuel identification algorithms. Unfortunately, this important component lacks in most of the reviewed literature. The inclusion of an IMU and GNSS information in the datasets enables a more efficient exploration and testing of sensor fusion algorithms and localization methods. By encompassing these different sensor modalities, our datasets become comprehensive resources for advancing research and development in various domains.

Alongside the sensor possibilities, there are additional requirements that must be fulfilled to ensure a competent working solution. Firstly, it is critical that the system can run continuously for a minimum of two hours so that longer expeditions and/or multiple consecutive datasets can be collected without recharging its batteries. Additionally, the software should be well integrated using a commonly used middleware for Robotics applications such as ROS (Robot Operating System), and the system should be able to endure high outdoor temperatures to allow working under most weather conditions. This includes considering appropriate cooling solutions to avoid the sensors to go beyond their maximum operating temperature. Other important requirements include modularity for adding, swapping, or removing sensors and processing nodes and real-time notification of sensor malfunctions during startup and runtime. These characteristics are mainly achieved through ROS and Docker—further explained later on—and highly improve the system’s robustness and potential for adoption in different scenarios. In addition, the apparatus should be affordable (below $4500), and its weight should be kept to a minimum so as not to cause discomfort to the user. Naturally, multiple sensors and a small-factor onboard computer must be incorporated to achieve the proposed goals, and all sensors must be kept fixed in the apparatus structure with a well-known geometrical relationship between them at all times.

**Figure 2:**
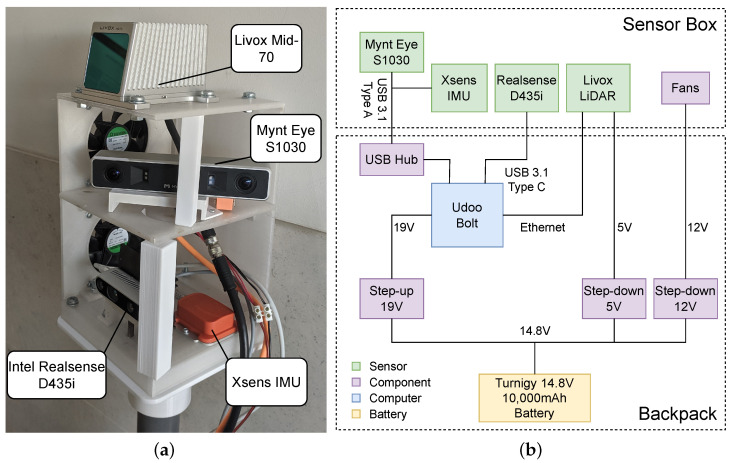
A closer look at the multiple sensors incorporated in the system and their connectivity and power management. (**a**) Sensor framework. (**b**) Physical system block diagram.

As shown in Figure 2a, the system comprises the following components: a Xsens MTi IMU, a Mid-70 Livox LiDAR, a Mynt Eye S1030 stereo camera, and an Intel Realsense D435i RGB-D camera. The LiDAR publishes point clouds at 50 Hz with precise measurements and a maximum range of 90 m. However, the LiDAR’s limited circular Field of View (FoV) of 70.4° restricts the amount of information it can capture. To complement the LiDAR data, the Mynt Eye provides point clouds with a larger horizontal FoV of 146°. The Intel Realsense D435i camera not only provides additional depth information but also serves as the single source of RGB and infrared information, which is useful for identifying relevant forest entities, such as flammable material [3]. The system also contains an onboard computer, the Udoo Bolt V3, that is responsible for receiving and recording data from every sensor. The onboard computer is equipped with a high-speed M.2 NVMe Solid-State Drive (SSD) to allow the simultaneous recording of high volumes of data acquired by the different sensors. The entire system is powered by a 14.8 V Turnigy battery with 10,000 mAh, which can provide approximately 4 h of continuous operation. For further clarification, a diagram showing how the various modules are physically interconnected and powered is presented in Figure 2b.

To ensure durability and functionality, the physical structure of the apparatus was divided into two distinct components: “sensor box” and “backpack”. The sensor box houses the sensors, while the backpack accommodates the computer, battery, and voltage regulators (cf. Figure 2b). The system is intended for use in outdoor environments, where ambient temperatures can reach over 35 °C for extended periods of time, and sensors experience increased heating. In such conditions, the use of some plastic materials such as Polylactic Acid (PLA) are not suitable due to their potential to deform or degrade. Given that the host computer can reach high temperatures, the structure inside the backpack is made of Acrylonitrile Butadiene Styrene (ABS), which can withstand temperatures of about 80 °C without significant degradation [42]. The sensor box, on the other hand, is built using polyethylene terephthalate glycol (PETG), which is a material that has a glass transition temperature at around 75 °C [43] but offers more adequate ultraviolet resistance, which is important considering that this is the most exposed part of the apparatus. To mitigate potential thermal issues in the sensor box, several fans are set up to blow fresh air into the cameras, which tend to heat up after long periods of operation. A heat sink is also attached to back of the Realsense D435i camera to improve heat dissipation. These measures not only help maintain optimal performance but also ensure that the temperature of the sensors remains within safe limits, minimizing any potential safety risks to the operator. Moreover, to facilitate future upgrades, the Livox LiDAR is mounted on top, allowing for easy replacement with a LiDAR with a larger horizontal FoV in the mid-term future. Since the primary purpose of the Mynt Eye is to increase the FoV for mapping, the mount that holds it in place was designed to allow easy rotation around the yaw axis. This enables users to adjust the extent to which the Mynt Eye’s data overlaps with the FoV of the LiDAR and the D435i as needed in their specific application.

Sensor poses are known in a common frame of reference from the Computer-Aided Design (CAD) of the system with the exception of the yaw angle of the Mynt Eye due to its adjustable nature. Sensor registration is completed manually using the transformations provided by CAD. Once the Mynt Eye is physically set by the user in its final orientation, its pose is passed as an input parameter of the system by visually comparing the 3D intersection of the different sensor point clouds. From our experience, this procedure yields appropriate results in general. Yet in the future, we intend to work on more precise and automated extrinsic calibration of the apparatus’ sensors.

**Figure 3:**
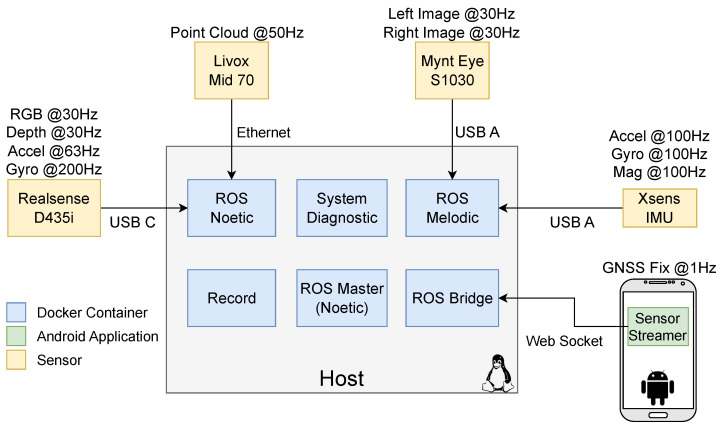
Complete system architecture, highlighting the different sensors, devices and Docker containers.

We use a software platform for packaging and running applications in isolated containers, since the sensor drivers run in different, not fully cross-compatible versions of ROS. Therefore, the project’s complete architecture for the dataset recording process, as depicted in Figure 3, is designed around Docker. It includes containers for the different ROS versions needed as well as a container running a ROS bridge server, which exposes port 9090 on the host computer to receive GNSS information from an in-house developed Android Sensor ROS application (see [5] for more information). Additionally, another container is responsible for writing the dataset into the rosbag format, and a debugging node runs on a System Diagnostic container, recording a separate rosbag dataset with various useful monitoring information, such as sensor acquisition frequencies and CPU temperature. The use of Docker also enables easy replication of the architecture for different apparatuses with sensor configurations specific to each use case. The codebase for the complete system architecture can be accessed at https://github.com/Forestry-Robotics-UC/fruc_dataset_apparatus, accessed on 12 June 2023.

## 5. Experimental Procedure

**Figure 4:**
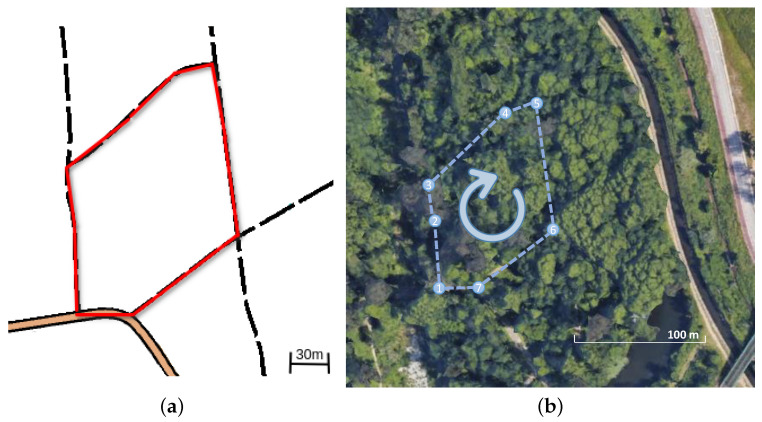
Aerial/top views of the navigated path at the experimental site in the Choupal National Woods. (**a**) Route map [44]. (**b**) Satellite view (Google Earth).

A dataset [4] was collected at the Choupal National Woods (40°13′13.3″ N; 8°26′38.1″ W) in Coimbra, Portugal, with the specific aim of evaluating the apparatus’ effectiveness in challenging outdoor conditions. The dataset was collected on a sunny day, where the user performed two circular loop laps, amounting to a distance of approximately 800 m (as depicted in Figure 4) with a duration of 15 min 33 s. Throughout the experiment, the smartphone collecting GNSS information was kept in a fixed position in relation to the apparatus, with the android application running in the foreground with the phone’s screen actively on. The phone was connected to a network carrier, and the cellular data plan was activated to improve the Assisted GNSS estimation. The woodland environment featured a diverse range of visual elements, including tree trunks, trees, bushes, and leaves, providing rich features for evaluation.

The dataset entails a diverse range of ROS sensor data acquired at different frequencies. The stored data includes IMU readings, i.e., 3D orientation, angular velocity and linear acceleration; magnetic field readings and its internal temperature values (all at 99.26 Hz). The Livox LiDAR provides 3D point clouds at 49.88 Hz, while the smartphone provides Assisted GNSS (A-GNSS) fix data (latitude, longitude, etc.) at 1.05 Hz. Left and right monochromatic stereo images are obtained by the Mynt Eye camera at 19.82 Hz, while the Intel Realsense acquires RGB and depth images at 29.68 Hz, together with accelerometer (63.33 Hz) and gyroscope (197.90 Hz) measurements from the internal IMU. The data captured offer a comprehensive view of the environment, enabling extensive analysis and facilitating research and development for numerous applications.

**Figure 5:**
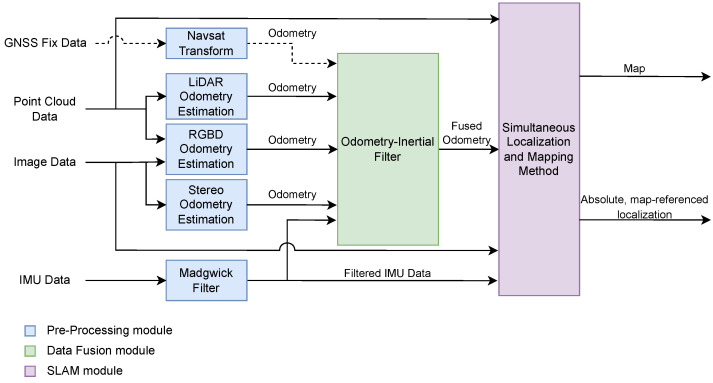
High-level diagram of the process to acquire a map of the environment and an absolute, map-referenced localization using the data from the available sensors. Dashed arrows represent optional connections.

In order to assess the quality of the collected dataset, we employ different SLAM methods to obtain reliable localization data. Localization plays a critical role in various applications that involve navigation, such as map building and transversability analysis. It serves as the foundation for perception-based algorithms to operate effectively, and therefore, it is paramount to provide localization information alongside raw data in a dataset to increase the potential use cases. In addition, SLAM algorithms can also serve as a valuable means to evaluate the quality of the dataset. For instance, if an RGB feature-matching algorithm in SLAM performs well and successfully maps and recognizes loop closures, it suggests that the images captured by the RGB-D camera possess sufficient quality for other algorithms like metric-semantic mapping. The same logic can be applied for the LiDAR scans. The utilization of various types of SLAM methods and the subsequent evaluation of their performance enables us to gauge the dataset’s overall quality and its suitability for a wide range of applications. This showcases the flexibility and usability potential of the dataset provided. For this, we focus on two prominent, distinct and proven open-source ROS-based SLAM algorithms: Cartographer and RTAB-Map. These are compared and evaluated based on a decoupled multimodal architecture, as illustrated in Figure 5.

Relevant raw data collected with the diverse sensors have been processed using dedicated ROS nodes to convert sensory information into odometry motion estimates. LiDAR odometry from the Livox Mid 70 is computed using the livox_mapping [45] ROS package, which extracts feature points from point clouds to obtain relative poses via frame matching and is especially tailored for the Livox LiDAR series.

Odometry estimation from the Intel Realsense D435i’s RGB-D images is obtained using rgbd_odometry, which is part of the RTAB-Map ROS package. The node computes visual features and depth information from depth images; then, it applies feature correspondences between images and Random Sample Consensus (RANSAC) to extract the most likely transformation between consecutive images. For stereo images from the Mynt Eye S1030 camera, we use the stereo_odometry node, which is also included in the RTAB-Map ROS package. The node computes visual features extracted from the left images with their depth information computed by finding the same features on the right images. Then, it also uses feature correspondences and a RANSAC approach to extract the most likely transformation between the consecutive left images.

GNSS Fix data can also be converted into an odometry input for late fusion with the remaining estimates of relative pose. For this, one can use the navsat_transform_node included in the robot_localization ROS package. In our architecture, we set this input as optional, given the limited capabilities of GNSS in the woodland scenario where the experiments were performed due to tall trees and large canopies that can obstruct the line of sight between the GNSS receiver and the satellites, and introduce multipath interference, thus leading to a degradation of GNSS positioning.

The odometry estimates derived are then fused with the IMU measurements. For this, the angular velocities, linear accelerations, and magnetic readings from the XSens MTi IMU are firstly fused using a Madgwick Filter (see [46] and imu_filter_madgwick ROS package). This provides a 3D orientation estimate that is passed on as an additional input together with the odometry estimates into the data-fusion module to provide a 6D fused odometry input (3D position and 3D orientation) to the SLAM method.

The fusion node uses an Unscented Kalman Filter implementation from [47], which is available in the robot_localization ROS package. The resulting fused odometry, along with sensor point clouds and filtered IMU measurements feed either the Cartographer or the RTAB-Map SLAM methods to generate a map and an absolute map-referenced localization.

It is important to note, however, that the dataset collected as described above is unlabeled and also lacks ground-truth reference localization information. Therefore, a set of quantitative and qualitative metrics need to be defined for algorithm evaluation and/or training. For SLAM, in particular, since in this experiment, the user’s start pose matches their final pose, the quantitative RTE and Relative Angular Error (RAE) metrics can be computed as:

(1) $$\mathrm{RTE}=\sqrt{{\left(x_{final}-x_{start}\right)}^{2}+{\left(y_{final}-y_{start}\right)}^{2}+{\left(z_{final}-z_{start}\right)}^{2}},$$

and

(2) $$\mathrm{RAE}=\sqrt{{\left(\phi_{final}-\phi_{start}\right)}^{2}+{\left(\theta_{final}-\theta_{start}\right)}^{2}+{\left(\psi_{final}-\psi_{start}\right)}^{2}},$$

where ϕ, θ, and ψ represent the Euler angles (roll, pitch, and yaw respectively).

To assess the alignment and consistency of the SLAM algorithm outputs with the real-world environment, a qualitative metric was employed. This metric involves overlaying the localization onto a map of the Choupal National Woods, which was generated by O-Solutions [44] through the human practice of cartography. This visual comparison provides clear insights into the alignment and consistency of the SLAM algorithms’ outputs with respect to the real-world environment.

## 6. Results and Discussion

The experimental evaluation results reveal distinct characteristics of the two SLAM methods tested. Cartographer and RTAB-Map employ different sensor modalities to estimate their respective localizations. RTAB-Map utilizes Odometry, IMU, and RGB-D information, while Cartographer leverages Odometry, IMU, and LiDAR data. The versatility of the recorded dataset and the apparatus becomes evident in supporting these diverse sensor configurations, showcasing its adaptability and effectiveness to accommodate SLAM approaches with distinct assumptions.

**Figure 6:**
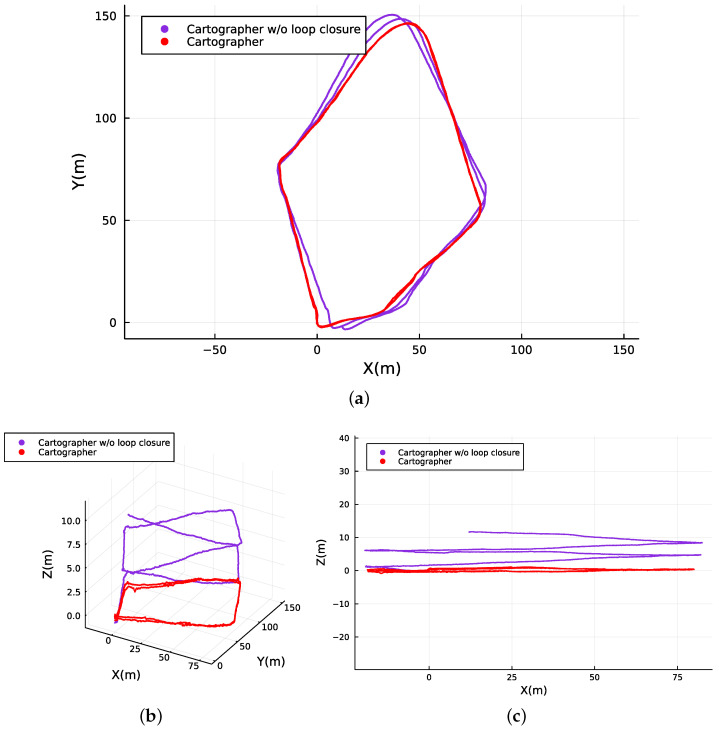
Absolute map referenced localization of the traveled path computed with and without loop closure by Cartographer. (**a**) Top view. (**b**) 3D isometric perspective. (**c**) Side view.

**Figure 7:**
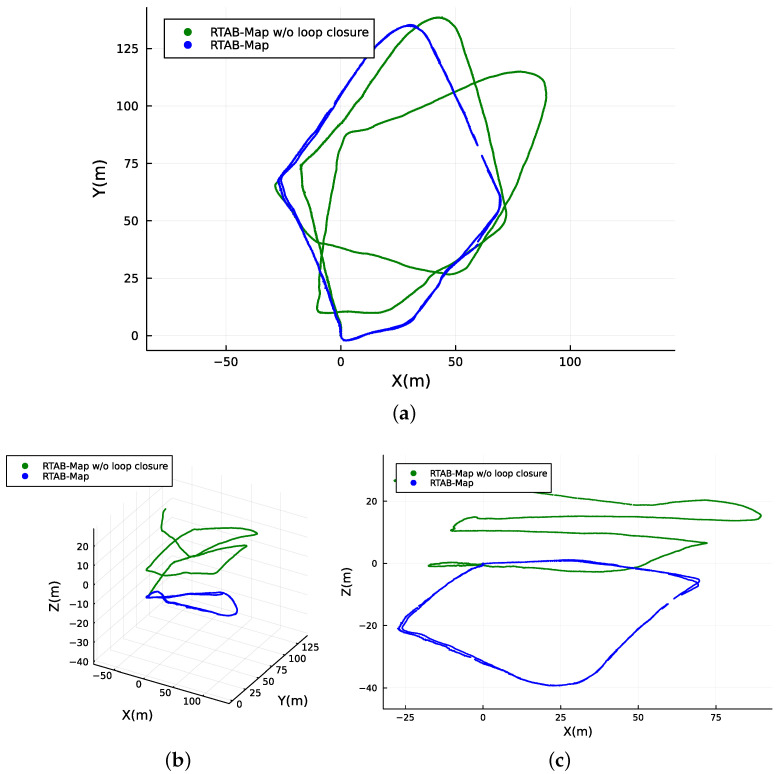
Absolute map referenced localization of the traveled path computed with and without loop closure by RTAB-Map. (**a**) Top view. (**b**) 3D isometric perspective. (**c**) Side view.

Figure 6 and Figure 7 illustrate the localization results of adopting the SLAM techniques along the navigated path followed by the user while carrying the apparatus on his back. It can be observed that both methods are able to continuously localize the system with different levels of success when applied to the data collected.

**Figure 8:**
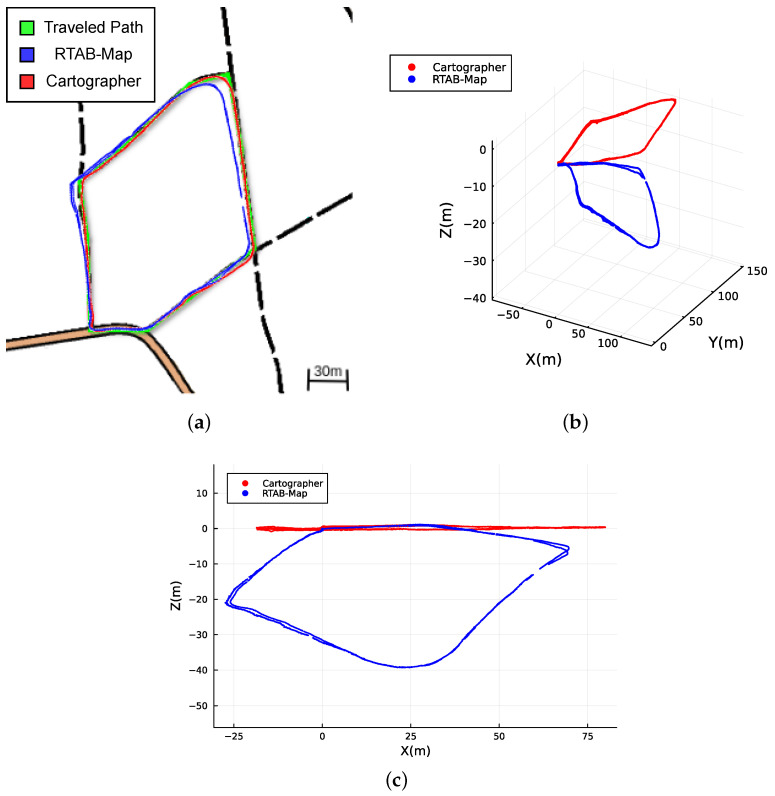
Absolute map referenced localization of the traveled path computed with loop closure by Cartographer and RTAB-Map. (**a**) Overlay of RTAB-Map and Cartographer localization with the route map of Figure 4a. (**b**) 3D isometric perspective. (**c**) Side view.

Since the methods are based on GraphSLAM, special attention is placed on loop closure identification and its impact on the overall results. RTAB-Map uses visual features for loop closure, while Cartographer is supported by LiDAR-based loop closure. Both approaches successfully identify loop closures along the path (see Figure 6 and Figure 7), which helps to significantly reduce the RTE and RAE for the two methods, as shown in Table 2. This indicates that the dataset has enough distinct features from multiple modalities for the SLAM implementations to recognize previously visited locations and enhance the overall mapping accuracy. However, the RTAB-Map with loop closure exhibits a noticeable negative variation along the Z-axis, despite the dataset being collected in a relatively flat terrain. This is not observed when RTAB-Map operates without loop closure, as illustrated in Figure 7c. This suggests that the Z-axis variation issue is primarily related to the back-end graph optimization of the SLAM algorithm. In woodland and forest scenarios, visual similarities and locations that look alike are common, which may cause false positives in loop closure. This can also be caused by sensor noise and/or lighting changes, and it is more likely to occur if visual features are used, as in the case of RTAB-Map. Moreover, we made use of the default graph optimization approach GTSAM [48] in RTAB-Map. Yet, it also supports TORO [19] and g2o [18]. We hypothesize that further tuning of the back-end parameters could eventually enhance the Z-axis variation and the overall trajectory accuracy for RTAB-Map. In contrast, Cartographer demonstrates higher localization precision, with the back-end successfully optimizing large sparse pose graphs with the Ceres Solver [49] while keeping the trajectory on leveled ground (refer to Figure 7c). As a result, it achieves superior overall localization performance as shown in Figure 8 when compared to RTAB-Map, overlaying accurately on top of the reference route map from [44], as illustrated in Figure 8a.

**Figure 9:**
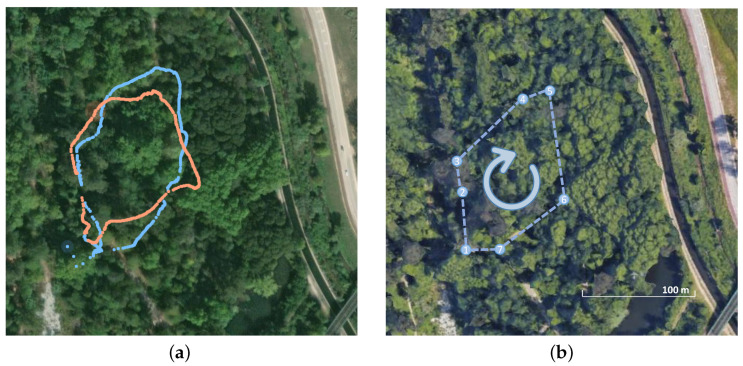
Assisted GNSS (A-GNSS) positioning from a smartphone integrated in the apparatus. (**a**) A-GNSS data during first (blue) and second (orange) laps of the navigated path (Google Maps). (**b**) Satellite view (Google Earth) of the navigated path. Replicated from Figure 4b for comparison.

The A-GNSS data recorded from the smartphone did not yield satisfactory results in terms of accuracy and trajectory representation, as illustrated in Figure 9. While the two loops followed by the user are discernible, the overall shape of the trajectory is not consistent with the navigated path. The woodland environment, with its tall trees and dense canopies, poses significant challenges for acquiring reliable GNSS data. The obstructed visibility of satellites in such an environment hampers the quality and reliability of the A-GNSS measurements. Even though the proposed architecture supports GNSS input and this is provided in the dataset for further study, recognizing the sub-optimal nature of these measurements, we have decided not to include them in the sensor fusion node. This decision avoids the potential degradation of localization performance in both SLAM algorithms, as the incorporation of unreliable data could introduce errors and inconsistencies into the fusion process.

**Figure 10:**
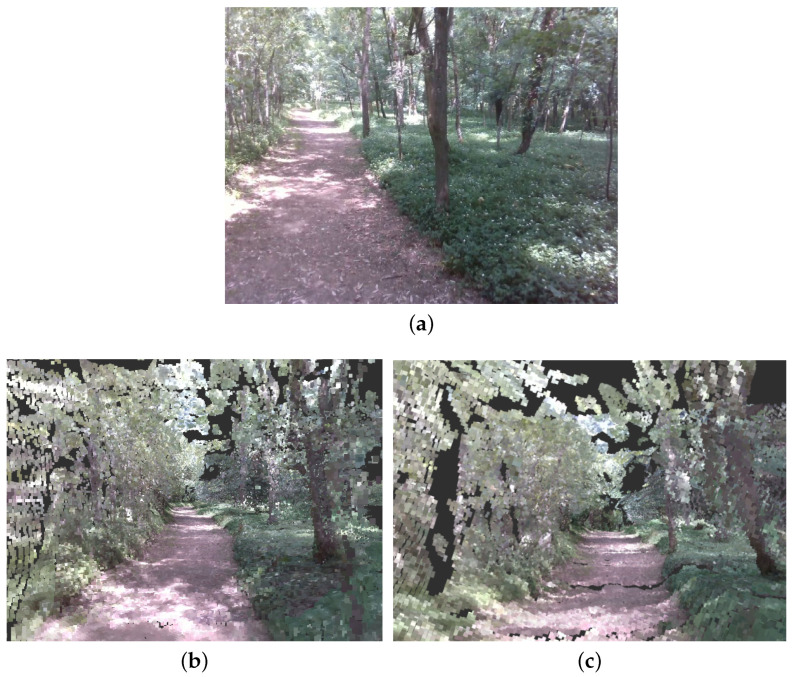
Octree maps generated by using each method’s localization with loop closure correction and the RGB-D outputs as inputs to the UFOMap package [53]. (**a**) Reference RGB image. (**b**) Scene reconstruction using Cartographer. (**c**) Scene reconstruction using RTAB-Map.

The maps built when executing the two SLAM methods are significantly different. RTAB-Map supports the generation of 3D point clouds [50] or a 3D octomap [51] of the environment, while Cartographer produces a 2D occupancy grid [52]. To allow for a side-by-side comparison, one can use the localization yielded by the SLAM method together with a mapping framework that reconstructs a comprehensive 3D RGB map utilizing the RGB-D information contained in the dataset. In this work, we employ these SLAM methods alongside the UFOMap 3D mapping framework [53] to build consistent and optimized 3D colored octree maps of the environment, as depicted in Figure 10. Comparing the generated maps with an image (Figure 10a) captured from a similar vantage point, it is evident that both maps exhibit consistency. The distinct features of the path are clearly discernible amidst the surrounding vegetation, including small bushes and grass, highlighting the accuracy and fidelity of the reconstructed map.

**Figure 11:**
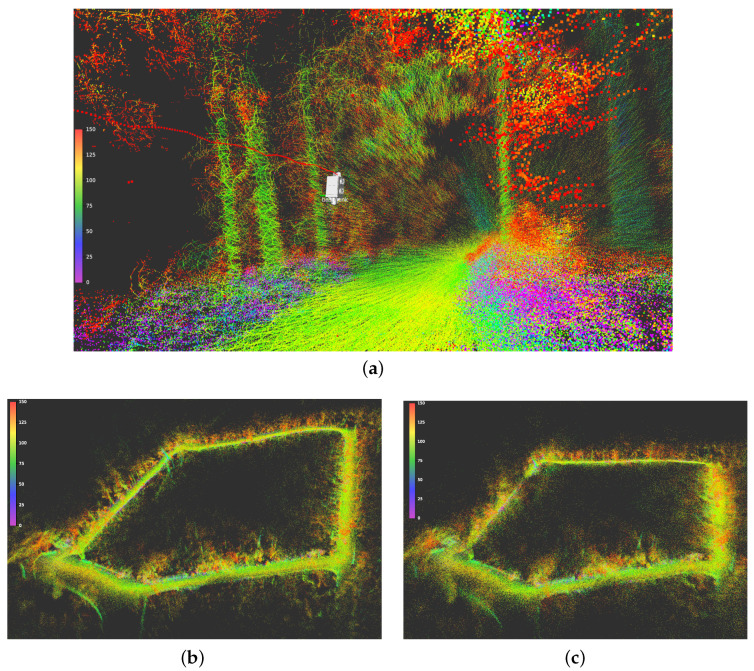
Livox LiDAR point clouds registered into a map using the 6D localization computed by Cartographer and RTAB-Map, with both methods using loop closure correction. In this representation, colors represent the LiDAR intensity, i.e., the strength of the backscattered echo at each point. (**a**) Close-up view of registered point clouds while traversing the navigated path with localization extracted from Cartographer. (**b**) 3D reconstruction of travelled path in isometric perspective using Cartographer localization. (**c**) 3D reconstruction of travelled path in isometric perspective using RTAB-Map localization.

In the above discussion, we have used the RGB-D camera data to build the 3D colored octree map of the environment. However, another representation of the environment can be accomplished by registering the colorless point clouds obtained from the 3D LiDAR sensor using the localization data derived from the SLAM methods. This is illustrated in Figure 11. This approach allows for the inclusion of more distant features in the map due to the extended range of the LiDAR. The resulting map is consistent and provides clear identification of salient features such as trees and the borders of the path. When examining the map from a close-up of a 3rd person view of the apparatus shown in Figure 11a, individual trees are reconstructed with a high level of detail, showcasing the dataset’s ability to capture fine details. Figure 11b,c provide isometric perspectives on the complete maps generated by the Cartographer and RTAB-Map localizations, respectively. Although both maps demonstrate consistency, it is evident that the Cartographer map exhibits significantly sharper details as the features on Figure 11c appear more blurred. A video of the data acquired with the generation of the maps using the dataset is available at https://youtu.be/9EXIwiExvWs, accessed on 16 June 2023. This detailed and precise representation of the environment enables researchers to gain valuable insights into the environment, facilitating tasks such as path planning and traversability analysis, and further analysis of the forest landscape, for instance through semantic segmentation or metric-semantic mapping.

**Figure 12:**
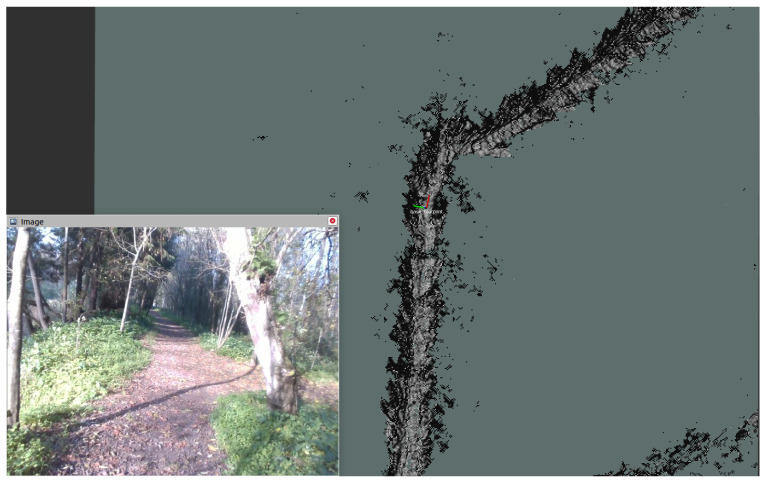
Mechanical effort costmap generated by the method presented in [54] with data from our dataset, in which lighter values in the grayscale represent easier to traverse areas and vice versa.

As a final, bonus example going beyond our use case scenario, Figure 12 shows the mechanical effort-based traversability technique proposed by Carvalho et al. [54] running with the data provided in our dataset. It uses point clouds to infer terrain gradient and the location of obstacles in space, and from there, it generates a global 2D costmap with mechanical effort information to guide the agent from one place to another in a way that minimizes the mechanical effort it is subject to and potentially its energy/fuel consumption.

The rich and accurate data extraction procedure also highlights the utility of acquiring multimodal datasets with the proposed apparatus, enabling a deeper understanding of the forest’s structural characteristics through robust perception capabilities.

## 7. Conclusions

In this study, we propose the development of a portable, lightweight and inexpensive apparatus for collecting multisensory data considering the requirements for forest and woodland environments while also allowing for the collection of datasets in any type of environment. Through experimental evaluation, providing insights into the performance of state-of-the-art SLAM implementations on the collected data, we demonstrate the versatility, feasibility and potential of the proposed approach in facilitating the planning, testing, and deployment of autonomous robots for forestry maintenance.

The dataset generated by the multi-sensor apparatus, openly available in [4], presents a contribution to the field of forestry robotics, as it provides the bulk of data required by researchers to analyze forest environments in depth, obtain an *a priori* map for robot operations, and label and train segmentation algorithms. The novel architecture developed for recording and storing the dataset provides a modular and user-friendly solution for acquiring extensive and dense datasets, seamlessly integrating into diverse platforms with various sensor combinations. Moreover, we also contribute to the community with an Android mobile app implementation, available in [5], which delivers GNSS/A-GNSS data for ROS systems out-of-the-box.

This opens up new possibilities for a more widespread adoption of autonomous robots in the field, improving the efficiency of data acquisition and reducing costs associated with automated vehicle deployment. Our study lays the foundation for future research and development in autonomous forestry maintenance, ultimately leading to safer and more efficient practices in forestry management.

Looking ahead, we plan to integrate a GNSS Real-Time Kinematic (RTK) station in our multi-sensor apparatus to facilitate reliable comparison between localization and/or SLAM algorithms. GNSS-RTK can deliver absolute gold standard positioning with centimeter-level precision, which is particularly valuable for localization-dependent algorithms, enabling more precise and refined results in these areas of research. Future datasets will be collected in various forest environments, with a particular focus on locations that have significant terrain variations. The current dataset lacks annotated images, but this limitation can be turned into an opportunity for users to apply their domain knowledge and expertise in annotating the images according to their specific needs, making the dataset more adaptable.

## Figures and Tables

**Table 1:** Comparison table between previously mentioned systems.

Work	IMU	LiDAR	GNSS	Stereo	RGB-D	SLAM Method	Error (m)	Forestry	Structure	^1^ Cost (USD)
Oveland et al. [8], 2018	✓	✓	✓	—	—	GeoSLAM (proprietary) [34]	N/A	✓	Backpack	$13,500
Proudman et al. [9], 2021	✓	✓	✓	—	✓	Factor-Graph LIO [35] + Elevation Mapping [36]	0.11 (RTE)	✓	Handheld	$19,000
Su et al. [10], 2021	✓	✓	—	—	—	Custom LiDAR-SLAM inspired by [37,38]	N/A	✓	Backpack	$9000
Jelavic et al. [2], 2021	✓	✓	—	✓	—	Cartographer [33]	0.41 (APE)	✓	Handheld	$10,500
Xiao et al. [39], 2022	✓	✓	✓	—	—	LIO-SAM [24]	0.03 (RTE)	—	Backpack	$10,000
Sier et al. [12], 2022	✓	✓	✓	✓	—	FAST-LIO [13] LIO-Livox [40]	0.05 (APE)	✓	Wheeled Cart	$43,000
Faitli et al. [14], 2023	✓	✓	✓	—	—	LIO-SAM-based [24]	0.02-0.16 (APE)	✓	Handheld	$34,000
Li et al. [15], 2023	✓	✓	✓	—	—	FAST-LIO [13] LOAM [41] LIO-LIVOX [40]	0.13-0.35 (APE)	✓	Helmet	$41,000
Our solution	✓	✓	✓	✓	✓	RTAB-Map [16] Cartographer [33]	0.09-0.28 (RTE)	✓	Backpack	$4000

^1^ The estimated costs outlined represent a lower bound value for the examined systems.

**Table 2:** Relative Pose Error (Translation and Rotation) of the SLAM methods tested at the same start and end pose.

Method	RTE (m)	RAE (rad)
RTAB-Map	0.089	0.25
RTAB-Map without loop closure	77.10	1.18
Cartographer	0.28	0.069
Cartographer without loop closure	17.10	0.21

## Data Availability

The data presented in this study are openly available in the Zenodo repository. DOI: 10.5281/zenodo.8139205.

## Abbreviations

The following abbreviations are used in this manuscript:

A-GNSS	Assisted Global Navigation Satellite System
ABS	Acrylonitrile Butadiene Styrene
APE	Absolute Position Error
CAD	Computer-Aided Design
CPU	Central Processing Unit
DBH	Diameter at Breast Height
FoV	Field of View
g2o	General Graph Optimization
GNSS	Global Navigation Satellite System
GPS	Global Positioning System
GraphSLAM	Graph-based Simultaneous Localization and Mapping
GTSAM	Georgia Tech Smoothing and Mapping
ICP	Iterative Closest Point
IMU	Inertial Measurement Unit
LIO	LiDAR-Inertial Odometry
LIO-SAM	LiDAR-Inertial Odometry with Smoothing and Mapping
LiDAR	Light Detection And Ranging
LeGO-LOAM	Lightweight and Ground-Optimized LiDAR Odometry and Mapping
LOAM	LiDAR Odometry and Mapping
PETG	Polyethylene Terephthalate Glycol
RANSAC	Random Sample Consensus
RGB-D	Red, Green, Blue and Depth channels/sensor
ROS	Robot Operating System
RTAB-Map	Real-Time Appearance-Based Mapping
RAE	Relative Angular Error
RTE	Relative Translation Error
RTK	Real-Time Kinematics
SC-LeGO-LOAM	Scan Context Lightweight and Ground-Optimized LOAM
SLAM	Simultaneous Localization And Mapping
SSD	Solid-State Drive
TORO	Tree-based netwORk Optimizer
UFOMap	Unknown Free Occupied Map
